# Hyperammonemic encephalopathy in a 46 year old patient treated with valproic acid as treatment for borderline personality disorder: a case report.

**DOI:** 10.1192/j.eurpsy.2024.1449

**Published:** 2024-08-27

**Authors:** P. Setién Preciados, E. Arroyo Sánchez, C. Díaz Mayoral, J. Gimillo Bonaque

**Affiliations:** Servicio de Psiquiatría, Hospital Universitario Príncipe de Asturias, Alcalá de Henares, Spain

## Abstract

**Introduction:**

Valproic acid (VPA) has been used in clinical practice since the 60’s, with a relatively favourable safety and efficacy profile. Pancreatitis, hepatotoxicity and teratogenicity are the most significant adverse drug reactions. VPA is also known for causing hyperammonemia, which may be asymptomatic or can present with encephalopathy. VPA-induced hyperammonemic encephalopathy (VHE) is a serious but reversible condition, which requires high clinical suspicion for diagnosis. It may occur acutely or after chronic use of VPA.

**Objectives:**

Review how frequent is for valproic acid to cause hyperammonemic encephalopathy, signs to watch out for and how it can be treated.

**Methods:**

Presentation of a patient’s case and review of existing literature, in regards to encephalopathy caused by valproic acid as a result of ammonia elevation.

**Results:**

In the case displayed here, the patient is diagnosed of hyperammonemic encephalopathy after being treated with valproic acid as treatment for borderline personality disorder.

Reviewing literature, cases of hyperammonemia are rarely reported as VPA-induced, probably because this increased level of ammonia in blood can vary between asymptomatic, and clinically relevant levels. Symptomatology due to VPA-induced hyperammonemia include: lethargy, impaired consciousness, focal neurological signs and symptoms and increased seizure frequency. More rare described symptoms are: aggression, ataxia, asterixis, vomiting and coma.

There are multiple treatment modalities for patients diagnosed with VHE, the primary treatment being the discontinuation of VPA. Other treatments frequently used are Lactulose and Carnitine.

**Conclusions:**

VHE is a rare occurrence, however can have fatal outcomes if not recognized and managed in time. Physicians should be vigilant while initiating Valproate therapy to patients. Clinicians should consider the possibility of VHE in patients with unexplained altered mental status, regardless of the duration of VPA therapy. A timely diagnosis is essential to prompt effective treatment, thus ensuring the patient’s safety and decreasing the length of hospitalisation and the cost of care in hospitals.

**Disclosure of Interest:**

None Declared

